# Pattern of HIV testing and multiple sexual partnerships among men who have sex with men in China

**DOI:** 10.1186/1471-2334-13-549

**Published:** 2013-11-16

**Authors:** Eric P F Chow, Jun Jing, Yuji Feng, Dai Min, Jun Zhang, David P Wilson, Xiaohu Zhang, Lei Zhang

**Affiliations:** 1 The Kirby Institute, University of New South Wales, Sydney, New South Wales, Australia; 2 Comprehensive AIDS Research Center, Tsinghua University, Beijing, China; 3 Melbourne Sexual Health Centre, Alfred Hospital, Melbourne, Victoria, Australia; 4 Central Clinical School, Faculty of Medicine, Nursing and Health Sciences, Monash University, Melbourne, Victoria, Australia; 5 Bill & Melinda Gates Foundation, Beijing Representative Office, Beijing, China; 6 China Food and Drug Administration Institute of Executive Development, Beijing, China

**Keywords:** Men who have sex with men (MSM), China, HIV testing, Sexual behaviours

## Abstract

**Background:**

Men who have sex with men (MSM) are a hidden but emerging population susceptible to HIV infection against a background of rapidly increasing HIV prevalence in China. Low HIV testing levels and multiple partnerships among MSM are two major contributing factors to HIV transmission.

**Methods:**

We conducted a cross-sectional survey among 447 Chinese MSM in Changsha and Tianjin cities from November to December 2011 using an anonymous questionnaire. We aim to investigate (1) the trend of HIV testing rates among Chinese MSM during 2009 to 2011; and (2) the patterns of multiple sexual relationships with male, female and commercial partners.

**Results:**

The self-reported past-12-months HIV testing level among Chinese MSM increased from 16.6% in 2009 to 46.3% in 2010 and 58.6% in 2011 (χ^2^ = 173.49, p < 0.001). Compared with men who have tested for HIV, the never-tested MSM were generally younger, never married, students, and more likely to have unprotected anal intercourse with non-commercial male partners. Furthermore, 21.3% (56/263) MSM reported having multiple regular male and female sexual partnerships and 6.2% (16/257) reported having commercial male partners in the past six months. However, individuals who were never-tested for HIV are consistently less likely to engage in multiple sexual relationships.

**Conclusions:**

HIV testing rates have increased substantially among Chinese MSM in the period 2009–2011, although significant barriers to testing remain. Multiple sexual partnerships, and especially bisexual behaviours, are common among Chinese MSM.

## Background

The predominant mode of HIV transmission in China has shifted in the last decade from sharing of injecting equipment among drug users to sexual transmission [1]. The epidemic has emerged and spread rapidly among men who have sex with men (MSM) in recent years [1-3]. The national prevalence of HIV among Chinese MSM has quadrupled from 1.4% in 2001 to 6.3% in 2011 [4,5] and the proportion of annual HIV incidence attributable to homosexual contacts increased from 12.2% in 2007 to 32.5% in 2009 [2]. Chinese MSM are a complex population, as the majority of MSM not only participate in homosexual activities [6] but also in frequent heterosexual activities and the commercial sex trade [6-8]. Multiple sexual partnerships with both men and women are common among Chinese MSM and approximately 70-90% will eventually enter heterosexual marriage [9-11]. Reported low condom use between MSM [12] and regular female partners [7] suggests a potential bridge of HIV infection to the general female population through their multiple sexual partnerships [13,14].

HIV testing is a primary and effective strategy to identify infections; early diagnosis of HIV also enables timely treatments in infected individuals [15]. Despite the recent report of substantial improvements, the national level of annual HIV testing rate among Chinese MSM (50.4% in 2011 [5]) remains substantially lower than other developed countries such as Norway (56%) and Australia (60-70%) [16,17]. In addition, mathematical modelling of HIV transmission among Chinese MSM suggests that 87% of HIV-positive MSM are not aware of their infection [18], indicating a very high undiagnosed rate compared with MSM in developed countries [15,19-21]. Understanding the underlying socio-demographic and behavioural contributing factors to HIV testing among MSM is crucial for effective prevention efforts.

It has become apparent that the patterns of HIV testing and multiple types of partnerships are two key but under-investigated areas among MSM in China. In the light of this, we conducted a retrospective cross-sectional study to investigate (i) the trend of annual and repeated HIV testing among MSM during 2009–2011; and (ii) their patterns of sexual behaviours with multiple types of sexual partners (non-commercial male, female and commercial male) in the past six months.

## Methods

### Study subjects and recruitment

A community-based cross-sectional study among MSM was conducted in Changsha and Tianjin cities from November to December 2011. Potential participants were recruited through venue-based convenience sampling and peer-referral. Peer recruiters were the volunteers from local MSM community-based organizations (i.e. ‘Tianjin Deep Blue Voluntary working group’ in Tianjin; ‘*Changsha Zonda-Sunlight Working Party*’ and ‘*Zuo An Cai Hong*’ in Changsha). Ten volunteers were trained for outreach to recruit participants in well-known MSM hotspots including gay bars, clubs, saunas and bathhouses. Each volunteer spent three to four hours on outreach recruitment on every weekend evening throughout the 2-month period. In addition, peer recruiters and participants were also asked to refer their peers to participate in this study. Participants were eligible if they were (i) aged 15 or over and had engaged in anal or oral sex with men in the past 12 months; and (ii) able to recall whether they had ever been tested for HIV in their lifetime. Participants were asked to complete an anonymous questionnaire of approximately 15–20 minutes duration through face-to-face interviews. All participants, if necessary, were invited or referred to the voluntary counselling and testing (VCT) service that offered by the local community-based organizations.

### Measures

The questionnaire covered aspects of demographic characteristics, HIV testing history and sexual behaviours (see Additional file 1). Study participants were asked about demographic characteristics including age, marital status (never married, married or cohabiting with female, divorced or widowed, cohabiting with male, and other), residency (local, and non-local), educational level (junior high school or below, senior high school, and college or above), occupation (student, self-employed, employed in state-owned or foreign companies, and other) and self-reported sexual orientation (homosexual, heterosexual, bisexual and unsure).

Questions related to HIV testing history and behaviours were stratified according to whether the participants had ever received an HIV test. In the case of negative responses, the participants were asked about the reason(s) for not having an HIV test in the past. A list of fourteen possible reasons was provided and participants could give more than one answer. Participants who indicated that they had ever had an HIV test were asked to recall the context and frequency of HIV testing in the previous three years (2009–2011). All participants were asked about their activity with various types of sexual partnerships (non-commercial male, commercial male and non-commercial female), including specific questions such as (i) whether they had had sex with different types of sexual partners in the past six months; (ii) condom use during the last anal/vaginal intercourse; (iii) awareness of HIV serostatus of the last sexual partner; and (iv) how long ago had the last three episodes of sexual intercourse occurred.

### Statistical analysis

Questionnaire data were double entered and checked in Microsoft Access 2007 (Microsoft Corporation, Washington, USA). Participants were categorised into three independent groups (tested within the past 12 months; tested more than 12 months ago; and never tested) [22] to investigate the differences between the three groups, in demographic characteristics and sexual risk behaviours. Descriptive statistics and frequency distribution of the sample were calculated. We employed Chi-Square test to investigate the association between the dependent variable and the categorical study variables; continuous study variables were tested by using one-way analysis of variance (ANOVA). Significance level of 0.05 was used for all statistical tests. Gap durations (i.e. number of days) between the last two consecutive episodes of sexual intercourse in different types of partnerships were also estimated. All statistical analyses were performed in STATA version 12.0 (StataCrop, Texas, USA).

### Ethical considerations

Verbal and written consent procedures were given to the study participants and they had the right to discontinue the survey at any time. This study was approved by the Human Research Ethics Committee of the University of New South Wales (HC12450) and the Institutional Review Board of the Tsinghua University (#00201204).

## Results

### Demographic characteristics

A total of 455 MSM were recruited in this study. Eight were excluded as they could not recall whether they had been tested for HIV. The remaining 447 participants met the eligibility criteria and completed the questionnaire; of these, 238 were from Changsha city and 209 from Tianjin city. Although significant differences in demographic characteristics were observed between the two groups (Table 1), HIV testing behaviours did not significantly differ (χ^2^ = 1.26, *p* = 0.53). Since the within-sample correlation of demographic characteristics with the testing behaviours were similar but with different compositions in two cities independently (see Additional file 2), data from the two cities were pooled together for the analyses of associated factors of HIV testing (Table 2). Of the 447 participants, 262 (58.6%) were tested within the past 12 months; 95 (21.3%) were tested more than 12 months previously; and 90 (20.1%) had never been tested. MSM who had never been tested were significantly younger (26.42 ± 8.64 years) than those who had tested within the past 12 months (27.88 ± 7.76) and more than 12 months ago (30.17 ± 7.85) (*F* = 4.80, *p* = 0.03). In terms of marital status, the majority of never-tested MSM (80.0%) had never married compared with those who had tested within the past 12 months (62.2%) and more than 12 months ago (54.7%) (χ^2^ = 16.88, *p* < 0.001). Furthermore, a significantly higher proportion of students had never tested for HIV compared with those who were employed (χ^2^ = 16.60, *p* < 0.001). There was no significant association between self-identified sexual identify and testing behaviours (χ^2^ = 1.35, *p* = 0.51). No significant association between HIV testing behaviours and residency of the study participants (χ^2^ = 3.39, *p* = 0.18) was observed.

**Table 1:** Socio-demographic characteristic and sexual behavioural factors that associated with HIV testing among Chinese MSM

**Characteristics**	**Total**		**Changsha**		**Tianjin**		**χ**^**2**^	***P-value***
	**(N = 447)**		**(N = 238)**		**(N = 209)**			
	***n***	***%***	***n***	***%***	***n***	***%***		
Age							6.80	0.03*
<20	30	6.7%	22	9.2%	8	3.8%		
20-39	361	80.8%	182	76.5%	179	85.6%		
≥40	54	12.1%	32	13.4%	22	10.5%		
Missing	2	0.4%	2	0.8%	0	0%		
Marital status							3.02	0.08
Never married	287	64.2%	142	59.7%	145	69.4%		
Married/Cohabiting/divorced/widowed	153	34.2%	89	37.4%	64	30.6%		
Others/Missing	7	1.6%	7	2.9%	0	0%		
Residency							20.97	<0.001***
Local	216	48.3%	89	37.4%	127	60.8%		
Non-local	222	49.7%	140	58.8%	82	39.2%		
Missing	9	2.0%	9	3.8%	0	0%		
Education Level							37.411	<0.001***
Junior high & lower	32	7.2%	9	3.8%	23	11.0%		
Senior high	141	31.5%	52	21.8%	89	42.6%		
College & above	273	61.1%	176	73.9%	97	46.4%		
Missing	1	0.2%	1	0.4%	0	0%		
Occupation							0.02	0.90
Student/Others	234	52.3%	124	52.1%	110	52.6%		
Employed	209	46.8%	112	47.1%	97	46.4%		
Missing	4	0.9%	2	0.8%	2	1.0%		
Self-identified sexual identity							16.05	<0.001***
Homosexual	353	79.0%	201	84.5%	152	72.7%		
Heterosexual/Bisexual	83	18.6%	27	11.3%	56	26.8%		
Unsure/Missing	11	2.5%	10	4.2%	1	0.5%		
HIV testing behaviour							1.26	0.53
Tested within the past 12 months	262	58.6%	145	60.9%	117	56.0%		
Tested more than 12 months ago	95	21.3%	49	20.6%	46	22.0%		
Never tested	90	20.1%	44	18.5%	46	22.0%		

Note: *p < 0.05, **p <0.01 and ***p <0.001.

**Table 2:** Socio-demographic characteristic and sexual behavioural factors that associated with HIV testing among Chinese MSM

	**Tested within the past 12 months**		**Tested more than 12 months ago**		**Never tested**		**χ**^**2**^	***P-value***
				
	**(N = 262; 58.6%)**		**(N = 95; 21.3%)**		**(N = 90; 20.1%)**			
	***n***	***%***	***n***	***%***	***n***	***%***		
***Demographic characteristics***								
Age							13.95	0.01**
<20	16	6.1%	1	1.1%	13	14.4%		
20-39	214	81.7%	80	84.2%	67	74.4%		
≥40	30	11.5%	14	14.7%	10	11.1%		
Missing	2	0.8%	0	0.0%	0	0.0%		
Marital status							16.88	<0.001***
Never married	163	62.2%	52	54.7%	72	80.0%		
Married/cohabiting/divorced/widowed	95	36.3%	43	45.3%	15	16.7%		
Others/Missing	4	1.5%	0	0.0%	3	3.3%		
Residency							3.39	0.18
Local	122	46.6%	43	45.3%	51	56.7%		
Non-local	134	51.1%	51	53.7%	37	41.1%		
Missing	6	2.3%	1	1.1%	2	2.2%		
Recruitment location							1.26	0.53
Changsha	216	82.4%	43	45.3%	51	56.7%		
Tianjin	222	84.7%	51	53.7%	37	41.1%		
Education Level							4.94	0.29
Junior high & lower	24	9.2%	4	4.2%	4	4.4%		
Senior high	76	29.0%	33	34.7%	32	35.6%		
College & above	162	61.8%	58	61.1%	53	58.9%		
Missing	0	0.0%	0	0.0%	1	1.1%		
Occupation							16.60	<0.001***
Student/Others	143	54.6%	34	35.8%	57	63.3%		
Employed	117	44.7%	61	64.2%	31	34.4%		
Missing	2	0.8%	0	0.0%	2	2.2%		
Self-identified sexual identity							1.35	0.51
Homosexual	213	81.3%	73	76.8%	67	74.4%		
Heterosexual/Bisexual	46	17.6%	22	23.2%	15	16.7%		
Unsure/Missing	3	1.1%	0	0.0%	8	8.9%		
***Homosexual behaviours (non-commercial)***								
Had sex with male partners in the past 6 months							3.18	0.20
No	17	6.7%	3	3.3%	8	10.0%		
Yes	237	93.3%	89	96.7%	72	90.0%		
Used condom during the last anal intercourse							25.10	<0.001***
No	70	29.7%	19	21.6%	41	56.9%		
Yes	166	70.3%	69	78.4%	31	43.1%		
HIV status of last sexual partners^^^							5.22	0.07
Unaware	127	53.8%	49	55.7%	49	69.0%		
Aware	109	46.1%	39	44.3%	22	31.0%		
*HIV-positive*	2	0.8%	2	2.3%	0	0.0%		
*HIV-negative*	107	45.3%	37	42.0%	22	31.0%		
***Homosexual behaviours (commercial)***								
Had commercial sex partners in the past 6 months							3.96	0.14
No	127	76.0%	41	80.4%	47	88.7%		
Yes	40	24.0%	10	19.6%	6	11.3%		
Used condom during the last anal intercourse							0.47	0.79
No	4	10.0%	1	10.0%	1	20.0%		
Yes	36	90.0%	9	90.0%	4	80.0%		
HIV status of last sexual partners^^^							N/A	N/A
Unaware	40	100%	5	100%	5	100.0%		
Aware	0	0%	0	0%	0	0%		
***Heterosexual behaviours***								
Had female sex partners in the past 6 months							0.52	0.77
No	127	75.6%	35	71.4%	41	77.4%		
Yes	41	24.4%	14	28.6%	12	22.6%		
Used condom during the last vaginal intercourse							3.85	0.15
No	20	50.0%	9	64.3%	9	81.8%		
Yes	20	50.0%	5	35.7%	2	18.2%		
HIV status of last sexual partners^^^							9.58	0.05*
Unaware	22	55.0%	2	14.3%	4	36.4%		
Aware	18	45.0%	12	85.7%	7	63.6%		
*HIV-positive*	0	0%	1	7.1%	1	9.09%		
*HIV-negative*	18	45.0%	11	78.6%	6	54.5%		
***Multiple partnerships in the past 6 months***								
Had both non-commercial male and female sex partners							1.01	0.60
No	130	79.3%	36	73.5%	43	81.1%		
Yes	34	20.7%	13	26.5%	10	18.9%		
Used condom with non-commercial male and with female sex partners during the last anal/vaginal intercourse							3.25	0.20
No	16	48.5%	8	61.5%	8	80.0%		
Yes	17	51.5%	5	38.5%	2	20.0%		
Had both non-commercial male and commercial male sex partners							5.48	0.06
No	127	78.4%	41	83.7%	49	92.5%		
Yes	35	21.6%	8	16.3%	4	7.5%		
Used condom with non-commercial and commercial male sex partners during the last anal intercourse							10.12	0.01**
No	4	11.8%	1	12.5%	3	75.0%		
Yes	30	88.2%	7	87.5%	1	25.0%		
Had both female and commercial male sex partners							4.50	0.11
No	145	88.4%	47	95.9%	49	96.1%		
Yes	19	11.6%	2	4.1%	2	3.9%		
Used condom with commercial male and with female sex partners during the last anal/vaginal intercourse							3.16	0.21
No	6	30.0%	0	0%	1	100%		
Yes	14	70.0%	2	100%	0	0%		
Had both non-commercial male, commercial male and female sex partners							6.16	0.05*
No	145	90.6%	47	95.9%	51	100%		
Yes	15	9.4%	2	4.1%	0	0%		
Used condom with non-commercial male, commercial male and female sex partners during the last sexual intercourse							0.70	0.40
No	4	26.7%	0	0%	0	0%		
Yes	11	73.3%	2	100%	0	0%		

Note: *p < 0.05, ** *p* <0.01 and *** *p* <0.001.

^Chi-square test was performed between MSM who were aware their partners’ HIV status (positive or negative) and those who were not.

### Trend, frequency and barriers of HIV testing

**Figure 1:**
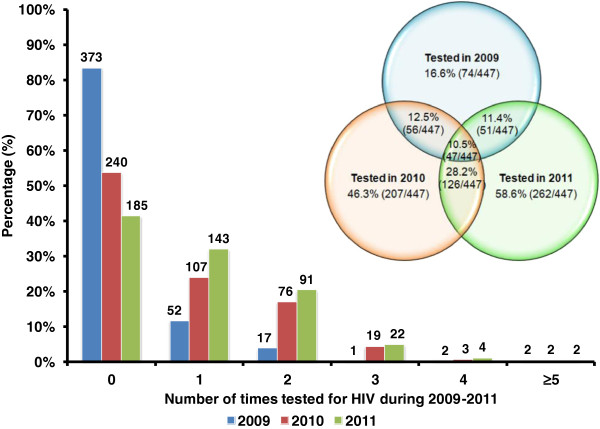
**HIV testing behaviours in the past 12 months among 447 MSM from 2009 to 2011.** The bar charts represent the number of HIV tests received per person per year. The venn diagram shows the proportion of consecutive HIV testing throughout the three-year period.

Figure 1 showed that the annual HIV testing rate among MSM significantly increased from 16.6% (74/447) in 2009 to 46.3% (207/447) in 2010 to 58.6% (262/447) in 2011 (χ^2^ = 173.49, *p* < 0.001). Among those who have been tested, a substantial proportion had been tested more than once in 2009 (29.7%, 22/74); 2010 (48.3%, 100/207); and 2011 (45.4%, 119/262). In total, 879 HIV tests had been conducted among 357 ever-tested MSM during 2009–2011. Of this group, only 47 MSM had received at least one HIV test annually throughout the three consecutive years. Additionally, there was a slight decline in HIV testing incidence from 2010 (38.4 cases per 100 population) to 2011 (34.1 cases per 100 population), but the change was not significant (χ^2^ = 1.46, *p* = 0.228). Approximately 8.1% (29/357) participants self-reported of not knowing their current HIV serostatus. Of these 29 MSM, 16 had tested more than once during this period.

Psychological and structural barriers to HIV testing among the ninety never-tested MSM are outlined in Table 3. The major psychological barriers were self-perception as healthy (55.6%); perceived low risk of HIV infection due to consistent condom use (30.0%); and the fear of discovering their own serostatus (27.8%). Other reasons such as only having had sex with healthy-looking partners (18.9%); fear of exposing their sexual orientation (17.8%); having sex with regular partners only (14.4%); and potential social stigma due to diagnosis of HIV/AIDS (11.1%) were also reported as psychological barriers. Structural barriers to HIV testing included not knowing where to get tested (33.3%), real-name registration testing (31.1%) and concerns about confidentiality (31.1%).

**Table 3:** Reasons for not having HIV testing among MSM

**Reasons for not having HIV testing**	**N**	%*
***Psychological barriers***		
Perceive myself are healthy	50	55.6%
Always have consistent condom use during any sexual acts	27	30.0%
Fear of discovering own HIV status	25	27.8%
Only have sex with people who look healthy	17	18.9%
Fear to expose my sexual orientation	16	17.8%
Have sex with regular partners only	13	14.4%
Diagnosis of HIV would lead to discrimination and psychological burden	10	11.1%
AIDS cannot be cured, cannot do anything if positive	1	1.1%
***Structural barriers***		
Don’t know where to go for HIV testing	30	33.3%
Need to provide real name for HIV testing	28	31.1%
Doubt about the confidentiality of Department of Health	28	31.1%
Time clash between working hours and HIV testing	5	5.6%
Attitudes of medical staff at testing sites are bad	1	1.1%
Other reasons	11	12.2%

*Data and percentage were obtained and calculated from 90 MSM who never had an HIV test.

### Multiple sexual partnerships

**Figure 2:**
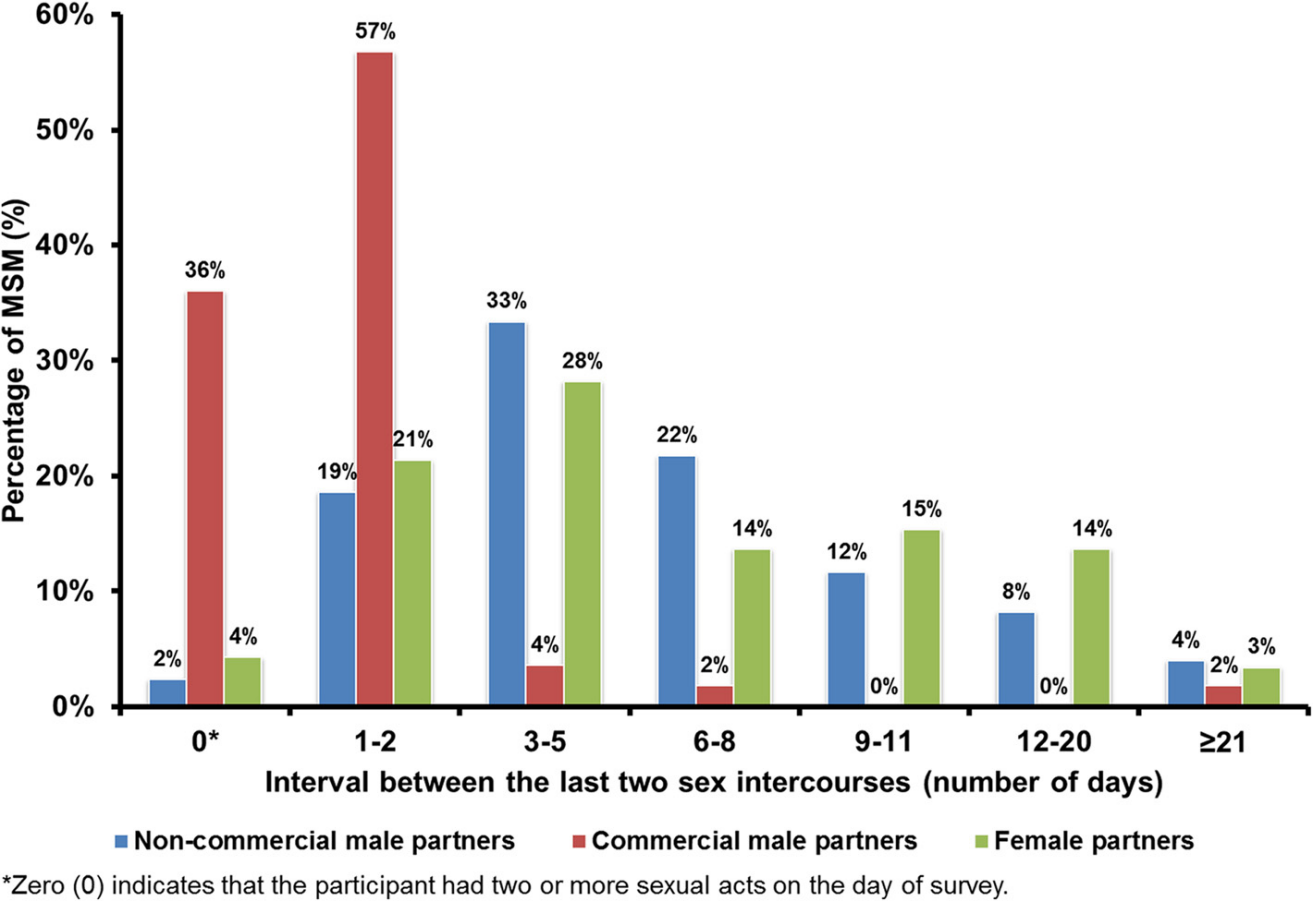
Distribution of sexual acts frequency between MSM with non-commercial male, commercial male and female partners.

Most MSM (93.4%, 398/426) had non-commercial male-to-male sex in the past six months. Our results indicated that the majority of never-tested MSM did not use condoms in the last sex act (56.9%), doubled the percentage of those who had tested in the past 12 months (29.7%) and those who tested more than 12 months ago (21.6%); the difference between three groups were highly significant (χ^2^ = 25.10, *p* < 0.001). Furthermore, less than half of ever-tested MSM (45.7%, 148/324) and never-tested MSM (31.0%) were aware of the HIV serostatus of their last non-commercial male partners. Among the 349 participants who reported non-commercial male-to-male sex, the average duration between the last two consecutive episodes of sexual intercourse with non-commercial partners was estimated to be 6.53 (95% CI 5.92-7.14) days (Figure 2).

About one-fifth of MSM (20.7%; 56/271) had male-to-male commercial (including both patronizing and selling) sex in the past six months. Higher levels of condom use with commercial sex partners were reported among both MSM who had tested in the past 12 months (90.0%, 36/40) and those who had tested more than 12 months ago (90.0%, 9/10), compared to those never-tested MSM (80.0%, 4/5); but the difference was not statistically significant (χ^2^ = 0.47, *p* = 0.79). Among the 56 participants who reported commercial male-to-male sex, all of them were ignorant of the HIV serostatus of their last commercial male sex partner. The duration between the last two consecutive episodes of commercial sex was approximately 1.17 (95% CI 0.61-1.74) days (Figure 2). Notably, more than one-third of the MSM (36.0%) had two or more episodes of commercial sex on the day of survey.

Our results showed that about 24.8% (67/270) of MSM had female sex partners in the past six months. Lower rate of condom use with female partners was reported among never-tested MSM (18.2%, 2/11) compared to those who had tested within the past 12 months (50.0%, 20/40) and those who had tested more than 12 months ago (35.7%, 5/14); however, the difference was not significant (χ^2^ = 3.85, *p* = 0.15). Significant association between HIV testing behaviours and the awareness of HIV serostatus of the last female sexual partner was observed (χ^2^ = 9.58, *p* = 0.05). Among the 62 participants who reported non-commercial sexual activity with a female partner, the interval between the last two consecutive episodes of heterosexual sex was 7.53 (95% CI 6.06-9.00) days (Figure 2).

**Figure 3:**
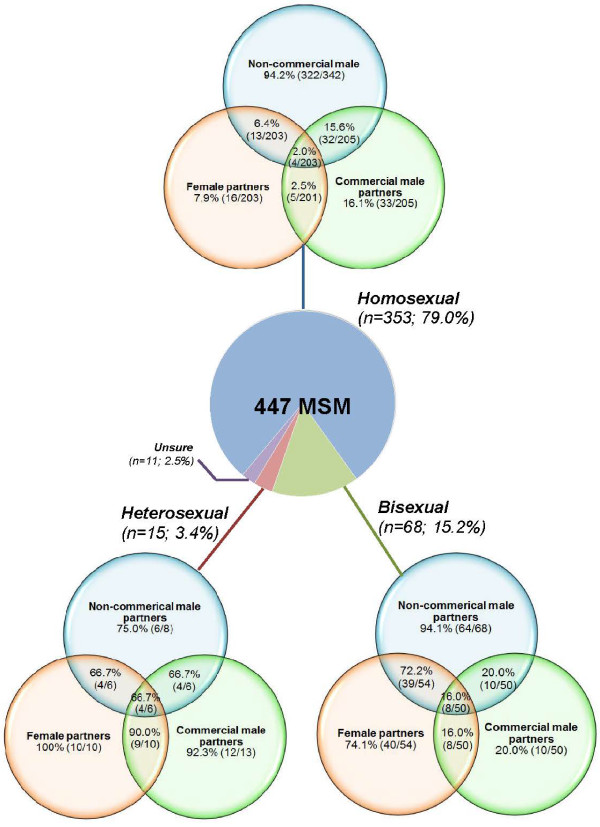
**Pattern of multiple sexual partners among Chinese MSM based on their self-identified sexual identity.** The bracketed nominator and denominator denote the number of individuals indicating an affirmative answer and the number of valid replies to the particular questions.

Figure 3 illustrates the multiple type of sexual partnership among MSM with different types of self-identified sexual orientation. Self-identified heterosexual (92.3%; 12/13) and bisexual (20.0%; 10/50) MSM were more likely to have commercial male partners in the past six months than self-identified homosexual men (16.1%; 33/205). Overall, about 21.3% (56/263) of MSM reported having both non-commercial male and female sexual partnerships and about 6.2% (16/257) of MSM reported having all three types of sexual partnerships in the past six months.

## Discussion

Our study demonstrates a significant increase in the HIV testing rate in the past 12 months among Chinese MSM from 16.6% to 58.6% during 2009–2011. The increase may be due to the ongoing expansion of the national ‘Four Frees and One Care’ program which provides free voluntary HIV screening tests [23]. Despite the increase in testing coverage, the proportion of MSM who test regularly remains extremely low, as only 13.2% (47/357) of ever-tested MSM have tested repeatedly for HIV in three consecutive years. Consistent with previous findings [24,25], MSM who are not aware their sexual partners’ HIV serostatus and those who have unprotected sexual intercourse at the last male-to-male sex act are more likely to take up HIV testing. In contrast, MSM who were younger, including those who are students or never married, are less likely to have been tested for HIV infection. Consistently, previous studies indicated that marital status may be a confounding factor of age [26,27], and the HIV higher testing rate among married MSM may be due to their more conservative perception of risk of HIV infection [28]. Among young students, insufficient HIV/AIDS knowledge and awareness due to inadequate sex education at school may contribute the low testing uptake [29]. As younger MSM are more sexually active and risk-taking in their sexual behaviours [30,31], intervention programs that target young MSM to improve HIV awareness and serostatus disclosure before and after entering marriage are essential.

Self-perception as ‘healthy’ or at ‘low risk of HIV infection’ are the main reasons for not having regular HIV testing in our study participants. This is consistent with findings in other large urban cities such as Beijing [24,28] and Shanghai [32]. The low-risk perception may explain the reported risk behaviours such as low condom use with male (43.1%) and female (18.2%) partners during the last sexual episode. Together with their low awareness of their sexual partners’ HIV serostatus, these behavioural patterns would substantially facilitate the effective transmission of HIV infection. Increasing perception of HIV risk and safe sexual practices remain a major task in HIV prevention among Chinese MSM [24].

Bisexual behaviours are common among our study participants as approximately one-fifth of the MSM (21.3%) reported having both male and female sexual partners in the past six months. This figure is slightly higher than previous studies conducted in Shanghai city (15-17%) [33,34] but is lower than in Shenzhen city (~38%) in Southern China [35]. Our result showed that older MSM are more likely to have entered heterosexual marriage and to have unprotected sex with their female partners [6,36,37], indicating the importance of HIV testing in preventing the spread of HIV through both homosexual and heterosexual partnerships. Among bisexual MSM, about 29.1% also participated in commercial sex with a male partner in the past six months. Bisexual MSM have sex with females as often as every 7.53 days, which is only slightly longer than the gap of homosexual intercourses with regular male partners (6.53 days). A meta-analysis showed that Chinese MSM with both male/female sex partners had significantly higher HIV prevalence than those with only male partners [38]. This strongly suggests that bisexual behaviours among MSM represent a very significant channel of HIV transmission to the female population [14].

Several limitations in this study should be noted. First, self-reporting and recall bias may have occurred in this retrospective study that requires recall of sexual behaviours in the past six months and the number of HIV tests received over the past three years. Participants might be inclined to provide socially desirable responses to the interviewers. Self-reporting bias may have occurred due to the varied demographic, ethnicity, level of education and culture. However, such association was not investigated in this study and hence caution is needed in interpreting results. In addition, the attitude and pattern of HIV testing could also be biased in different recruitment venues [39]. Second, as this study was conducted through both convenience and snowball sampling methods in two Chinese major cities, the mixed sampling method may affect the representativeness and ability to generalise from the sample to the broader MSM population in China.

Our study has several important implications for public health interventions and policies in China. First, scaling-up HIV testing efforts among younger MSM is a priority for effective HIV prevention among Chinese MSM. As this sexually active subgroup enters heterosexual marriage, bisexual behaviour of these individuals is likely to pose a significant risk in transmitting HIV infection to the low-risk female population [36,40]. Second, health promotion that aims to reduce psychological and structural barriers to HIV testing should be expanded among Chinese MSM. In addition to the traditional approaches of distributing educational materials through peer educators and at MSM hotspots, the internet has become an effective channel in disseminating relevant health information [22]. HIV pre- and post-test counselling provides unique opportunities to reduce the misconceptions about HIV infection and stigmatisation against people living with HIV (PLHIV) [41]. Third, recent meta-analyses indicated that behavioural interventions could effectively increase the uptake of HIV testing among Chinese MSM, but the majority of these interventions were only implemented in HIV-prevalent Southwest China [42,43]. Similar intervention programs could be expanded to other Chinese regions. Fourth, a substantial proportion (8.1%) of ever-tested study participants had not been notified of their test results. Strategic positioning of new HIV testing sites may substantially increase accessibility, and the roll-out of rapid HIV testing would assist in MSM receiving immediate screening results [28]. Furthermore, the use of innovative and proved approaches (i.e. automated SMS [short message service], computer pop-up reminders and alerts) may also increase the return for repeated HIV testing and timely notifications of test results [44,45].

## Conclusions

HIV testing rate has increased among Chinese MSM but remains relatively low in comparison to those in developed countries settings. In addition, multiple sexual partnerships with both male and female partners among Chinese MSM may facilitate HIV transmission in MSM community and into the general female population. Specific recommendations have been made, including scaling up HIV testing campaigns and health promotion interventions, in order to increase the coverage and reduce barriers of HIV testing among Chinese MSM.

## Competing interests

All authors declare that they have no competing interest.

## Authors’ contributions

EPFC participated in the study design, undertook the statistical analyses, wrote the first draft of the manuscript and was the primary author of this manuscript. JJ participated in the study design and revised the manuscript. YF obtained funding, critically reviewed and revised the manuscript. DM obtained funding and involved in the study design. JZ involved in the study design. DPW assisted in the study design and interpretation of results, critically reviewed and revised the manuscript. XZ participated in the data collection. LZ led the study, provided oversight in the study design and data analyses, critically reviewed and approved the final version of the manuscript. All authors contributed to and have approved the final version of the manuscript.

## Pre-publication history

The pre-publication history for this paper can be accessed here:

http://www.biomedcentral.com/1471-2334/13/549/prepub

## Supplementary Material

Additional file 1 **Questionnaire.** HIV testing behaviour among MSM in China. Click here for file

Additional file 2: Table S1 Socio-demographic characteristic factors that associated with HIV testing among Chinese MSM, stratified by cities (a) Changsha, and (b) Tianjin. Click here for file
