# Supplementary material for: Pattern of HIV testing and multiple sexual partnerships among men who have sex with men in China
Source: BMC Infect Dis. 2013 Nov 16;13:549. doi: 10.1186/1471-2334-13-549 (PMC3840637; doi:10.1186/1471-2334-13-549)
Supplement: Additional file 1 — Questionnaire. HIV testing behaviour among MSM in China. [file 1471-2334-13-549-S1.pdf]

Additional File 1. Questionnaire.

HIV testing behaviours among men who have sex with men in China

SECTION A
SAMPLE COLLECTION
(To be completed by the investigator)

A01 Questionnaire Number (0001-9999): □□□□
A02 Referral Number: (0001-9999): □□□□
A03 Date of investigation: □□/□□/□□□□
A04 Location: ① Changsha ② Tianjin

SECTION B
DEMOGRAPHICS CHARACTERISTICS

B01 Age: _______

B02 Marital Status:
① Single ② Married/Cohabiting with a female
③ Divorced/widowed ④ Cohabiting with a male ⑤ Others

B03 Residence: ① Local ② Foreign

B04 Education level:
① Primary ② Junior High ③ Senior High ④ College or above

B05 Occupation
① Student ② Self-employed ③ Civil ④ Foreign company ⑤ Others

B06 Sexual Orientation
① Homosexual ② Heterosexual ③ Bisexual
④ Unsure ⑤ Refuse to answer

SECTION C
HIV TESTING BEHAVIOURS

C01 Have you tested for HIV? ① Yes (Jump to C03) ② No (Jump to C02)
③ Can't remember (End)

C02 Reasons of not having HIV testing (can choose more than one item):
□ (a) They believe they are healthy
□ (b) Don't know the location of HIV testing sites
□ (c) Fear of having HIV
□ (d) Attitude of medical staff was not nice
□ (e) Diagnosis would lead to discrimination and psychological burden
□ (f) Had consistent use of condom
□ (g) Have regular sexual partners

☐ (h) Doubt about the confidentiality of department of health
☐ (i) Had sex with health partners only
☐ (j) Exposure of sexual orientation
☐ (k) AIDS cannot be cured
☐ (l) Time clash between working and HIV testing
☐ (m) Need to use real name for testing
☐ (n) Others

C03 When was your last HIV testing? Year ☐☐☐☐

	2011		2010		2009	
	**C04** Did you test for HIV?<br>① Yes<br>② No (Jump to C06)<br>③ Can't remember		**C06** Did you test for HIV?<br>① Yes<br>② No (Jump to C08)<br>③ Can't remember		**C08** Did you test for HIV?<br>① Yes<br>② No (Jump to C10)<br>③ Can't remember	
	**C05** Where did you have testing?		**C07** Where did you have testing?		**C09** Where did you have testing?	
Organizations	No. of testing	No. of testing with result noticed	No. of testing	No. of testing with result noticed	No. of testing	No. of testing with result noticed
Peer referral and testing campaign						
Rapid tested organized by NGO						
Mandatory tested in hospital or STD clinics						
Voluntary tested in hospital						
Tested at CDC clinics						
Blood donation						
Others						

C10 Are you HIV positive? ① Yes ② No (Jump to D01) ③ Don't know (Jump to D01)

C11 How long did you know you were infected by HIV? □□ Year □□ Months

C12 Are you currently receiving Antiretroviral Treatment? ① Yes ② No

SECTION D
SEXUAL BEHAVIOURS

	Male partners	Selling sex to male	Female partners
Had sex in the past six months?	**D01** ① Yes ② No (Jump to D05)	**D05** ① Yes ② No (Jump to D09)	**D09** ① Yes ② No (End)
Had condom use at last sex act?	**D02** ① Yes ② No	**D06** ① Yes ② No	**D10** ① Yes ② No
What was the HIV status of your last sexual partner?	**D03** ① Positive ② Negative ③ Don't know	**D07** ① Positive ② Negative ③ Don't know	**D11** ① Positive ② Negative ③ Don't know
Last three sex act occurred how many days ago?	**D04** ________, ________, ________,	**D08** ________, ________, ________,	**D12** ________, ________, ________,

Signature of investigator: [ ]

Signature of supervisor: [ ]

~ END OF QUESTIONNAIRE ~
