# Supplementary material for: Pattern of HIV testing and multiple sexual partnerships among men who have sex with men in China
Source: BMC Infect Dis. 2013 Nov 16;13:549. doi: 10.1186/1471-2334-13-549 (PMC3840637; doi:10.1186/1471-2334-13-549)
Supplement: Additional file 2: Table S1 — Socio-demographic characteristic factors that associated with HIV testing among Chinese MSM, stratified by cities (a) Changsha, and (b) Tianjin. [file 1471-2334-13-549-S2.pdf]

Additional File 2.

Table S1. Socio-demographic characteristic factors that associated with HIV testing among Chinese MSM, stratified by cities (a) Changsha, and (b) Tianjin.

(a) Changsha

	**Tested within the past 12 months (N = 145; 60.9%)**		**Tested more than 12 months ago (N = 49; 20.6%)**		**Never tested (N = 44; 18.5%)**		***F* or χ^2**	***P-value***
Characteristic	*n*	*%*	*n*	*%*	*n*	*%*		
Age (mean±SD)								
<20	12	8.3%	1	2.0%	9	20.5%	14.73	0.01**
20-39	110	75.9%	38	77.6%	34	77.3%		
≥40	21	14.5%	10	20.4%	1	2.3%		
Missing	2	1.4%	0	0.0%	0	0.0%		
Marital Status							28.68	<0.001***
Never married	79	54.5%	23	46.9%	40	90.9%		
Married/cohabiting/ divorced/widowed	62	42.8%	26	53.1%	1	2.3%		
Others/Missing	4	2.8%	0	0.0%	3	6.8%		
Residency							3.60	0.17
Local	59	40.7%	13	26.5%	17	38.6%		
Non-local	80	55.2%	35	71.4%	25	56.8%		
Missing	6	4.1%	1	2.0%	2	4.5%		
Education Level							3.66	0.45
Junior high & lower	7	4.8%	0	0.0%	2	4.5%		
Senior high	30	20.7%	10	20.4%	12	27.3%		
College & above	108	74.5%	39	79.6%	29	65.9%		
Missing	0	0.0%	0	0.0%	1	2.3%		
Occupation							21.50	<0.001***
Student/others	74	51.0%	16	11.0%	34	23.4%		
Employed	71	49.0%	33	22.8%	8	5.5%		
Missing	0	0.0%	0	0.0%	2	4.5%		
Self-identified sexual identity							0.71	0.70
Homosexual	126	86.9%	42	29.0%	33	22.8%		
Heterosexual/Bisexual	17	11.7%	7	4.8%	3	2.1%		
Unsure/Missing	2	1.4%	0	0.0%	8	5.5%		

Additional File 2.

(b) Tianjin

	Tested within the past 12 months (N = 117; 56.0%)		Tested more than 12 months ago (N = 46; 22.0%)		Never tested (N = 46; 22.0%)		*F* or χ^2	*P-value*
Characteristic	*n*	*%*	*n*	*%*	*n*	*%*		
Age (mean±SD)								
<20	4	3.4%	0	0.0%	4	8.7%	10.62	0.03*
20-39	104	88.9%	42	91.3%	33	71.7%		
≥40	9	7.7%	4	8.7%	9	19.6%		
Missing	0	0.0%	0	0.0%	0	0.0%		
Marital Status							1.19	0.55
Never married	84	71.8%	29	63.0%	32	69.6%		
Married/cohabiting/ divorced/widowed	33	28.2%	17	37.0%	14	30.4%		
Others/Missing	0	0.0%	0	0.0%	0	0.0%		
Residency							6.61	0.05*
Local	63	53.8%	30	65.2%	34	73.9%		
Non-local	54	46.2%	16	34.8%	12	26.1%		
Missing	0	0.0%	0	0.0%	0	0.0%		
Education Level							4.88	0.30
Junior high & lower	17	14.5%	4	8.7%	2	4.3%		
Senior high	46	39.3%	23	50.0%	20	43.5%		
College & above	54	46.2%	19	41.3%	24	52.2%		
Missing	0	0.0%	0	0.0%	0	0.0%		
Occupation							5.98	0.05*
Student/others	69	59.0%	18	39.1%	23	50.0%		
Employed	46	39.3%	28	60.9%	23	50.0%		
Missing	2	1.7%	0	0.0%	0	0.0%		
Self-identified sexual identity							0.99	0.61
Homosexual	87	74.4%	31	67.4%	34	73.9%		
Heterosexual/Bisexual	29	24.8%	15	32.6%	12	26.1%		
Unsure/Missing	1	0.9%	0	0.0%	0	0.0%		

Note: *p < 0.05, ** p <0.01 and *** p <0.001.
